# Disaster preparedness kits ready or not? Household resilience to flash flooding in Uttarakhand

**DOI:** 10.1016/j.heliyon.2024.e41446

**Published:** 2024-12-26

**Authors:** Praveen Maghelal, Sudha Arlikatti

**Affiliations:** a Faculty of Resilience, Rabdan Academy, PO Box 114646, Abu Dhabi, United Arab Emirates; b Amrita School for Sustainable Futures, Amrita Vishwa Vidyapeetham, Amritapuri Campus, Kerala State, India

**Keywords:** Disaster preparedness kits, Emergency kits, Uttarakhand flash floods, Household preparedness

## Abstract

The State of Uttarakhand, located in the Himalayan region of North India, suffered one of the worst flash floods on June 16, 2013 where 5700 were presumed dead or missing. Since then, the region has suffered two other major flooding events in 2021 and 2023. Although numerous national government agencies recommend keeping disaster preparedness kits ready, the saliency and effectiveness of a household disaster preparedness kit in helping survivors, has not been empirically tested in the region. Responses to “items they took with them when they evacuated for the flash flood” from 316 households were analysed. Majority were not prepared for evacuation with essentials like food, water, hygiene kits and items for children and the aged, and totally dependent on first responder agencies to come to their rescue. Our findings help to underscore the need to: 1) Ensure disaster kits are recommended by every Panchayat for local at-risk communities and handed to visitors/tourists at points of entry. 2) Revive the National School Safety Programme (NSSP) to help school children create disaster preparedness kits. 3) Conduct additional empirical research in various disaster contexts around the country to demonstrate the benefits for immediate survival and increasing household resilience.

## 1 Introduction

The State of Uttarakhand, located in the Himalayan region of North India, suffered one of the worst disasters in its history, the flash floods of June 16, 2013 where 5700 were presumed dead or missing [1]. A majority of the dead were Hindu pilgrims visiting the state from other parts of the country to complete their annual pilgrimage *(the Char Dham Yatra),* a tour of the four holy Hindu sites in Yamunotri, Gangotri, Kedarnath and Badrinath, situated in Uttarkashi District, part of the rugged terrain of the Himalayas in Uttarakhand. Since then, the region has suffered two other major flooding events in 2021 and 2023, and witnessed countless losses to lives and property. Located in the fragile ecosystem of the Himalayan ranges, the region is susceptible to seismic activities, flooding, erosion, landslides and earthquakes. With majority of the population, dependent on local economy, are observing increased urban growth and increased tourism, the risk of exposure to future events makes it evident to study the preparedness to flooding in the region.

Numerous national government agencies including the United States of America's Federal Emergency Management Agency [2] and the Government of India's National Disaster Management Authority [3] recommend that at-risk populations get better prepared by maintaining a household emergency preparedness kit. Humanitarian agencies like the International Federation of Red Cross and Red Crescent Societies [4] is also advising at-risk households to also set aside a shelter kit ready with two sets of tarpaulin and a shelter tool kit with nails, hammer and other essentials, so that they do not wait for government and aid agencies to keep their families safe. They can start looking for safe spaces and build a temporary shelter and keep their family in dignity while waiting for formalized assistance.

This is to ensure that vulnerable communities can survive the first 48–72 h on their own, before first responders or humanitarian agencies can reach them to provide food, shelter and other essentials. The items in these kits are context and hazard specific and may include drinking water, dry/packaged food, battery operated radios, torch, important documents, cash, female hygiene products, baby food and diapers, blankets, medical kit, medicines for chronic illnesses, batteries for cell phones, battery operated radio, nails, plastic sheets, tape, ropes, and important numbers of family and officials.

However, thus far the saliency and efficacy of a household disaster preparedness kit, has not been empirically tested except, for a study by Heagele [5] in the USA. While majority of the studies have assessed the use of emergency kits on preparedness and recovery of those affected [6,7], the determinants of household's preparedness kit have been less investigated. This study is aimed at filling this gap in the context of a hazard prone mountainous region of India, by examining what the populations who survived the 2013 flash floods in the state of Uttarakhand took with them as they evacuated. A joint team of US and Indian researchers conducted surveys of 316 respondents from 17 impacted villages from the lowlands, midlands, and uplands enroute to the Kedarnath temple in Uttarkashi [*for an enumeration see* [[1], [8]]]. The primary objective of this study is to assess what emergency kit items were most commonly taken by the respondents and what are the determinants of individuals choice to take an emergency kit item. Also, if they did, what characteristics related to their decision to take all the emergency kit items that they took during their evacuation.

While the context of the study being a decade old can be debated, there are several reasons why the outcome of this study is still valid. First, the number of events of flash flood has since 2013 increased gradually over the years in the state of Uttarakhand from 22 events (2015–16) to 84 events in (2021–22) [9]. Second, although the state disaster management authority in their action plan [10] discusses the use of emergency kit, the approach is in *response* to the disaster not as *preparedness* to mitigate the effect of flash flooding (pg. 128). Third, at the most the policy is to encourage families to carry emergency kit during evacuation (pg. 124), which indicates less initiative to prepare the communities through increased awareness and actual delivery of the kits, especially in wake of increased flash floodings in UK. The actual delivery of emergency kits has primarily been done by the private and non-profit entities such as the United Way [11], and Plan International [12]. While the public entities are yet to actively engage the communities for increased awareness and the supply of emergency kits, this study is critical for policy makers to understand the awareness and the ability of the families to prepare for future flash floods in the region.

Therefore, this study assessed the survey questions related to items that respondents took with them when they evacuated due to the flash flood, were analysed against their prior hazard experience, risk perceptions, socio-demographic characteristics, and location. Findings highlight the inhibitors to preparedness and underscore the need for better education efforts by the local population, and government entities to recognize the importance of disaster preparedness kits for helping survivors in this region be better prepared against future threats. The implications are specifically focused on the role of local government and emergency preparedness agencies to enhance the resiliency of the region by providing educational sessions, community training, emotional support and active participation in response to flooding in future.

## 2 Effectives and challenges of use of emergency kits

### 2.1 Effectiveness

In recent years, the frequency and intensity of flash flooding events have increased due to the impacts of climate change, posing a significant threat to communities worldwide. These rapid and localized floods can lead to devastating consequences, including loss of life, damage to infrastructure, and disruption of critical services. The development and deployment of effective emergency kits have become a crucial component in mitigating the risks associated with these extreme weather events.

Flash floods are defined as violent and short-lived floods generated by intense storms, which are particularly damaging due to their multidisciplinary nature and difficulty in forecasting [13]. These events can be caused by heavy rainfall associated with severe thunderstorms, hurricanes, or tropical storms, as well as the collapse of natural or man-made dams [14]. The risk of flash floods is particularly high in arid and semi-arid regions, which cover approximately one-third of the world's land surface [15].

Emergency kits are essential for mitigating the risks associated with flash flooding events [6]. The availability and accessibility of these kits can significantly improve the ability of individuals and communities to respond effectively to flash flooding incidents, reducing the impact on life, health, safety, infrastructure, and property [14]. Furthermore, emergency kits can play a crucial role in the recovery phase, as they can provide the necessary resources for individuals and communities to begin the rebuilding process [7].

Despite the numerous benefits of emergency kits, there are also several challenges and limitations that must be addressed. These include issues related to the cost and distribution of kits, the long-term sustainability of the kits, and the need for continuous education and training on their proper use. Additionally, the effectiveness of emergency kits may be limited by factors such as the severity and duration of the flash flooding event, as well as the availability of emergency response resources in the affected area [16]. By providing immediate access to essential supplies and resources, these kits can significantly improve the ability of individuals and communities to respond effectively to these catastrophic events, reducing the loss of life and property [[17], [18], [19]]. While studies have emphasized the effectiveness of emergency kits in resiliency, it is essential to address the challenges and limitations of emergency kits to ensure their long-term effectiveness and sustainability. Especially, the determinants and barriers of household's choice to carry emergency kit items during evacuation is less investigated.

### 2.2 Barriers

There may be a range of barriers that dissuade at-risk populations from purchasing and stockpiling supplies and preparing a ready to go emergency kit. These include an underestimation of risk by the people due to socio-economic, cultural factors and geographical factors, leading to low-risk perceptions (Bubeck et al., 2012), or denial and optimism bias for new risks [[20], [21], [22]] which leads some people to have “notions of invulnerability” and “unrealistic optimism”, preventing them from adopting preparedness measures. Kasperson et al. [23] suggested that oftentimes the technical assessment of risk as well interactions with the psychological and socio-cultural perspectives, and prior disaster experiences may amplify or attenuate public responses to the risk.

In some instances, extremely vulnerable populations including ethnic minorities, the elderly or people with disabilities who despite acknowledging the risks, may not have access or be able to afford to stockpile necessary supplies [24]. There are other unique transient populations like migrant workers and tourists and visitors, to the region who are also vulnerable due to their lack of knowledge or awareness of the inherent hazards or threats in the and hence do not know what preparedness actions to take and when to take them [[24], [25]]. However, effects of prior disaster experience on willingness to prepare are mixed, with some individuals who have experienced disasters firsthand not willing to engage in additional preparedness behaviors, because they feel adequately prepared due to their prior experiences [26] and others may be more willing to take preparedness measures if they experienced injury or deaths firsthand.

Recently, Sutton et al. [27] discussed the public warning systems proposed by Mileti and Sorensen [28] and suggested that information overload from multiple channels and stakeholders on disaster preparedness may overwhelm some individuals, leading to feelings of confusion on what to include in an emergency kit and apathy. On the other hand, communication gaps due to lack of a robust early warning system, and inadequate risk communication mechanisms by authorities, in remote locations, may hinder preparedness. Studies report inadequate preparedness leads to disorganized evacuation behaviour that puts the individuals and households at higher risk during evacuation (See [29,30]). Understanding and improving communication across various decision-making stakeholder and through different media outlets can provide better recommendations for effective preparedness to emergencies [31]. Therefore, increased awareness through appropriate communication channels (media and authority) can enhance disaster preparedness.

Several theoretical frameworks have been recommended to assess preparedness of communities against natural disaster. One such framework that has gained prominence among researchers if the Protective Action Decision-Making Model (PADM) proposed by Lindell and Perry [26]. This study uses the PADM framework to assess the exposure, awareness, perceptions and attention to the decision-making process to imminent threat. Using the responses to the items individuals carried during evacuation, this study examines the relation of various concepts identified in the framework on the decision-making of the respondent.

Consequently, this study investigates the individual's awareness to prepare for flooding in the at-risk mountain communities of North India. Broadly, we examine what people believe to be most important to carry when they evacuate and if this knowledge can be expanded to include better household preparedness. Specifically, this study investigates: (1) What items are considered critical by the evacuees, with little to no awareness and prior experience, of a flash flood? (2) What determinants are significant predictors of an individual carrying at least one of the emergency kit items? and (3) What determinants are significant predictors of carry any of the emergency kit items?

## 3 Methods

### 3.1 Study area

**Fig. 1:**
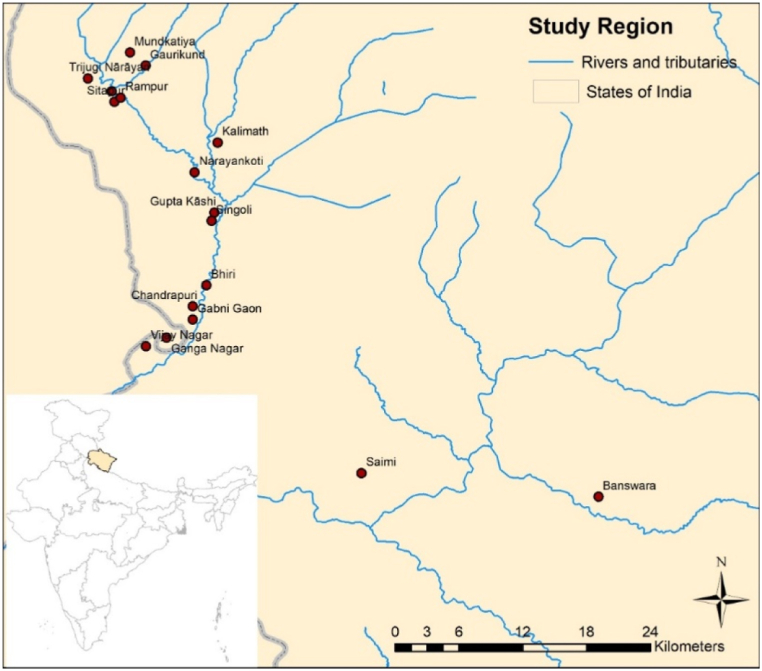
Location of villages.

The State of Uttarakhand, located in the Himalayan region of North India, suffered one of the worst disasters in its history on June 16, 2013. Almost the entire state received heavy rainfall leading to flash floods and landslides. Five of the thirteen districts namely, Bageshwar, Chamoli, Pithoragarh, Rudraprayag, and Uttarkashi were severely affected. A list of affected villages from the Uttarkashi district from the lowlands, midlands, and uplands, situated enroute to the Kedarnath temple were identified with the help of a local liaison officer. Of these 17 villages were selected, as sites to conduct surveys with villagers, in October 2013, four months after the flash floods (*see* Fig. 1). A convenient sampling strategy was adopted to identify respondents following which the local liaisons and village leaders helped to locate others directly impacted by the flooding as additional respondents.

### 3.2 Survey questionnaire

A total of 316 respondents provided information about the physical, social and household contexts in which people found themselves during the flood, perceptions of flood intensity, sources of threat information (environmental and social cues and social warnings), warning message channels and content, warning confirmation behaviour, risk perceptions, evacuation preparations, evacuation impediments, evacuation transportation modes, evacuation destinations, and shelter locations. Specifically, the questionnaire inquired about the demographics (age, gender, education status, length of residency in the region, and housing status), whether the individual lived in the flood plain, hazard communication experience (meetings and brochures), previous flash flood experience, traditional knowledge, pre-impact emergency preparedness, family flood damage and casualties, and other socio-cultural questions specific to the study area. Ethics approval for the study was obtained from the Institutional Review Board (IRB) at the University of North Texas and the participant consent was collected before the administration of the survey. The survey questionnaire was reviewed and edited for spellings, words, expressions, and then translated to Hindi (local language) and pre-tested for its clarity and local appropriateness.

### 3.3 Dependent variable

The number of items the respondents carried when evacuating from their residents after the flash flood hit was used as the dependent variable for this study. The responses were binary (Yes = 1; No = 0) for each of the item in the list and were assessed as sum of 12 items. The list of 12 items is consistent with the literature and the emergency management agencies across the globe [2,3]. Specifically, they were asked if they carried water, food, medicines, cash, change of clothes, female hygiene products, cell phone/radio, shelter destination address, important documents, LPG (liquid petroleum gas) cylinders for cooking, flashlights and blankets.

As reported in Table 1, majority of the respondents carried their cell phone/radio (41 %) followed by the address of a safe location (30 %), cash (22 %) and change of clothes (21 %). Surprisingly only 8 % reported carrying any medication, while even fewer carried a flashlight (3 %), blankets or important documents (1 %). It is likely that abundance of fresh water sources from springs and snowmelts in the region resulted in only 16 % reporting carrying drinking water, while 18 % reported carrying non-perishable food.

**Table 1:** Emergency Kit Items carried by the respondents.

Emergency Kit Items	Mean	Std. dev.	Min	Max
Water	0.16	0.37	0	1
Non-perishable Food	0.18	0.38	0	1
Medicines	0.08	0.28	0	1
Cash or Savings	0.22	0.42	0	1
Change of Clothes	0.21	0.41	0	1
Female Hygiene Products	0.06	0.24	0	1
Cellphone/Radio	0.41	0.49	0	1
Address of Safe Location	0.30	0.46	0	1
Important Documents	0.01	0.11	0	1
LPG Cylinder	0.02	0.12	0	1
Flashlight	0.03	0.18	0	1
Blankets	0.01	0.08	0	1

**Fig. 2:**
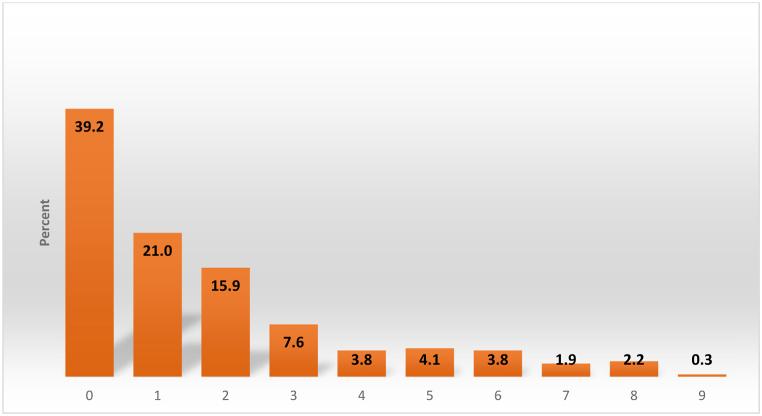
Number of emergency items carried by respondents.

Of the total respondents, 39 % did not carry any emergency kit items listed (*see* Fig. 2). While the median reports at least one-item, the mean indicates just over one item (1.69) was carried by the respondents. See Table 2 for an enumeration. In all, over 75 % of the respondents carried two or less (including no items) with them as they evacuated. This indicates either insufficient lead time to take appropriate items, or a lack of understanding of how long they would be away, and which items would be necessary for their immediate survival. It can also be attributed to little awareness on household preparedness and response to flash floods as none of them had experienced one before.

**Table 2:** Alignment of Critical Barriers to the survey questions.

No	Critical Barriers	Concept	Aligned Question	Measure
1	People lack the knowledge of how best to prepare	Awareness	Before the flash flood and landslides on June 16–17th 2013 had you ever attended any flash flood hazard awareness meetings?	Yes - 1; No - 0
2	People lack the means to purchase and maintain necessary supplies	Affordability	What is your family's monthly income range?	1 - Below Rs. 4999 2 - Between Rs. 5000 to 10,000 3 - Between Rs. 11,000 to 25,000 4 - Between Rs. 26,000 to 40,000 5 - Between Rs. 41,000 to 55,000 6 - Above Rs. 56,000
3	Household emergency preparedness information is often written at a too advanced literacy level	Education	What is the highest education level you have completed?	1 - Illiterate 2 - Less than 9th grade 3 - 10th pass (Secondary School Certificate) 4 - 12th pass (Higher Secondary School certificate) 5 - Some college 6 - Some certificate 7 - Bachelor degree 8 - Graduate or professional degree
4	People do not believe that they are at risk (e.g., for death, injury, property damage, and disruption to daily activities)	Risk Perception	To what extent did you believe the flash flood would do each of the following?	Responses to the following were summed (Each ranging from 1-not at all to 3-Very great extent) and grouped into low Risk, Medium Risk and High Risk: severely damage or destroy your home? injure or kill you or your family? disrupt your job and prevent you from working? disrupt electrical, telephone, and other basic services? destroy or severely damage many homes in your village/town? injure or kill many people in your village if they did not evacuate? place your life and the lives of your family at risk if you did not take precautionary measures and evacuate from where you were?
5	People already living in survival mode cannot be convinced to prepare for an event that may never happen	Pessimist	To what extent did you feel each of the following emotions during the floods?	Sum of all responses to: Depressed, Annoyed, Nervous, Fearful and Passive. Each ranging from 1-not at all to 3-Very great extent.
6	People have an unrealistic sense of optimism, do not take threats seriously, are in denial, or feel subjectively prepared but are not actually prepared objectively	Optimist	To what extent did you feel each of the following emotions during the floods?	Sum of all responses to: Optimistic, Relaxed, Energetic, Alert. Each ranging from 1-not at all to 3-Very great extent.
7	People are medically frail	Special Needs Individuals	Did any member of your household have special needs and require assistance to evacuate?	No - 1; Yes - 2
8	Household emergency preparedness information demonstrates a lack of consensus	Decision-making	Houeshold with individual over 65 years of age	Results from recent study by authors report the elders in the HH are usually not inclined to engage in preparedness

### 3.4 Independent variables

A study by Heagele [5] reviewed the available evidence in support of the effectiveness of emergency kits used in household preparedness to disasters. While the study argued that there is a lack of consensus on the exact composition of the emergency kits, the study identified 14 major barriers to prepare an adequate emergency kit (*see* 5). To understand the probability to take all possible items of an emergency kit, we aligned the barriers identified by that study to the survey responses from the current study. In all eight of the 14 barriers aligned with the questions and have been used as predictors of the outcome of this study (***see*** Table 2). The six barriers that did not align with the survey question included individual's inconvenience to maintain necessary supplies, availability of adequate space, ability to effectively mitigate risks, inaccessibility to kit during disaster, understand information provided only in English, and lack of access to internet.

The awareness of the individuals was minimal before the flash flood event (0.02; 1 indicating complete awareness) with majority in low-income category (1.97; Income between 5 and 10K) and education below high school (3.22; 3 indicating 10th pass) (*see* Table 3). While positive emotions were close to the median (8.08) the negative emotions were considerably high (12.02) which also relates to the higher mean risk perception of 2.13 (2 being medium risk perception). Households with individuals with special needs and elderly were low (1.17 and 0.35 respectively).

**Table 3:** Descriptive analysis of dependent and independent variables.

Variable	Obs	Mean	Std. dev.	Min	Max
Number of Emergency Kit Items	314	1.69	2.08	0	9
Awareness of Flash Floods	316	0.02	0.14	0	1
Income	316	1.97	1.31	1	6
Education	315	3.22	2.29	1	8
Positive Emotions	316	8.08	2.33	4	12
Negative Emotions	316	12.02	2.18	5	15
Perception of Risk	316	2.13	0.79	1	3
Household with special needs	314	1.17	0.38	1	2
Household with Elderly	316	0.35	0.64	0	2

### 3.5 Analysis

In the base scenario where individuals have little understanding of what constitutes an emergency kit, the two major research questions that motivates this study are: (1) does the individual's self-awareness of carrying an emergency preparedness kit vary from those unaware that they were carrying an emergency supply item, and (2) what barriers are significant predictors of individuals choosing to take an emergency kit item (binary response of yes or no), and (3) what barriers are significant predictors of individuals carrying any or all of the emergency items irrespective of their self-awareness (sum of all the items carried). Test of association was performed to analyse the first research question. Probit regression was performed to understand the likelihood of individuals choosing to take an emergency kit item while a negative binomial regression was performed to predict the number of items (count) taken be each household. With binary outcomes (yes/no) of choosing emergency kit item are not, the probit regression assumes that the latent variable has a normal distribution of errors for the outcome variable and hence appropriate for this analysis. Also, since the variance of the outcome variable is higher than the mean, the negative binomial regression model was selected over the Poisson regression model and has been identified as a better model to predict count-based outcomes to account for the over dispersion of the outcome variable [32].

## 4 Results

### 4.1 Self-awareness: Emergency kit or not?

Respondents were asked “what actions did you take before you evacuated from your location?”. One of the options was ‘gathered some emergency supplies’ which was measured as 1- yes, 0 – No. Only about 18 % (n = 58) of the total respondents reported carrying an emergency supply. While the 257 respondents reported not carrying any emergency supplies. When asked, “which of these materials (list of items of emergency kit, Table 1) did you have at hand when the flash flood hit?”, many of them reported carrying one or more of the emergency kit items. It should be noted that while some thought they carried an emergency supply with them, the items they carried are not among the key items of an emergency kit. The following lists only the key items of an emergency kit and its distribution (Table 4).

**Table 4:** Difference in self-awareness of respondents carrying emergency kit items.

Emergency Kit Items	Percent Carrying the Item		Percentage Difference	Probability
	YES (n = 58)	NO (n = 257)		
Water	27.6 % (16)	13.2 % (34)	14.40 %	0.007
Non-perishable Food	41.4 % (24)	12.4 % (32)	29.00 %	0.000
Medicines	19.0 % (11)	5.8 % (15)	13.20 %	0.001
Cash or Savings	44.8 % (26)	17.4 % (45)	27.40 %	0.000
Change of Clothes	43.1 % (25)	15.6 % (40)	27.50 %	0.000
Female Hygiene Products	12.1 % (7)	5.0 % (13)	7.10 %	0.047
Cellphone/Radio	57.0 % (33)	37.2 % (96)	19.80 %	0.006
Address of Safe Location	60.3 % (35)	22.9 % (59)	37.40 %	0.000
Important Documents	3.5 % (2)	0.8 % (2)	2.70 %	0.100
LPG Cylinder	5.2 % (3)	0.8 % (2)	4.40 %	0.015
Flashlight	0 % (0)	3.9 % (10)	−3.90 %	0.128
Blankets	0 % (0)	0.8 % (2)	−0.80 %	0.501

Note: Number of respondents in parenthesis.

Table 4 reports the percent of respondents who carried each of the emergency kit items grouped as those aware of carrying an emergency supply (n = 58 – *aware group*) and those that were not aware (n = 257 – *unaware group*). Besides carrying blankets, flashlight, and important documents, all other items reported a significant difference between the two groups. Most individuals in the aware group carried the address of safe location (60 %), cell phone (57 %), cash (45 %), change of clothes (43 %) and non-perishable food (41 %). While the unaware group reported highest percent for the same emergency kit items, they reported a significantly lower percentage with the highest difference observed for taking address of a safe location (37.4 %). This indicates that while the unaware group respondents were taking emergency supplies, it was still much lower in comparison to the aware group. The significant difference across the nine items indicates that self-awareness, in cases of individuals with no prior flash flood experience, can be an important motivator to carrying items critical for survival in emergency situations.

### 4.2 Carried an emergency kit item or not?

Responses to the list of items carried by each respondent was summed and categorized into two groups---Individuals who DID NOT CARRY any item were coded as ‘0’ and those who CARRIED ONE OR MORE items were grouped as ‘1’. A total of 39 % respondents reported not carrying any items.

The predictors of barriers to carrying of an emergency kit item or not were assessed using probit regression model (p = 0.0001) (***see*** Table 5). High income (>Rs. 56K) reported a negative relation (β = −0.88; p < 0.05) to taking an emergency kit item while higher levels of education, bachelor's degree (β = 0.83; p < 0.01) or graduate degree (β = 0.69; p < 0.10) reported a significant positive relation to carrying an emergency kit item. The smaller p-values of bachelor's degree and high income indicate stronger evidence against the null hypothesis of carrying an emergency kit item or not. Also feeling negative emotions (β = 0.09; p < 0.05), higher risk perception (β = 0.18; p < 0.10), and awareness of taking an emergency supply (β = 0.42; p < 0.00) reported a positive association with taking an emergency kit item during evacuation from the flash flood. Also, the confidence intervals of high income (>56K; CI: 1.65, −0.12), bachelor's degree (CI: 0.27, 1.38), negative emotions (CI: 0.01, 0.16) and taking emergency supplies (CI: 0.23, 0.60) are entirely negative or positive, not including a zero, indicating the range of values that likely contain the true effect size. On the contrary, the graduate degree (CI: 0.08, 1.46) and the perception of risk (CI: 0.02, 0.39) includes zero, meaning the effect size could be weak or null.

**Table 5:** Results of Probit Regression of carrying emergency kit item or not.

Carry Emergency Kit (Yes/No)	Coefficient	Std. error	z	P > z	[95 % conf.	interval]
Awareness of Flash Floods	−0.49	0.75	−0.66	0.512	−1.95	0.97
Income						
Between rs.5000–10,000	−0.02	0.20	−0.09	0.932	−0.41	0.37
Between rs.11,000–25,000	0.31	0.29	1.05	0.293	−0.27	0.88
Between rs.26,000–40,000	−0.36	0.34	−1.06	0.289	−1.03	0.31
Between rs.41,000–55,000	−0.39	0.46	−0.83	0.404	−1.29	0.52
Above rs. 56,000∗	−0.88	0.39	−2.26	0.024	−1.65	−0.12
Education						
Less than 9th grade	0.18	0.21	0.82	0.410	−0.24	0.60
10th pass SSC	0.00	0.25	−0.02	0.985	−0.50	0.49
12th pass HSC	0.28	0.29	0.99	0.324	−0.28	0.85
Some college	–					
Some vocational certificate	–					
Bachelor's degree∗	0.83	0.28	2.91	0.004	0.27	1.38
Graduate or professional degree∗	0.69	0.39	1.76	0.078	−0.08	1.46
Positive Emotions	0.05	0.03	1.51	0.130	−0.02	0.12
Negative Emotions∗	0.09	0.04	2.22	0.027	0.01	0.16
Perception of Risk∗	0.18	0.11	1.72	0.085	−0.02	0.39
Household with special needs	0.10	0.21	0.48	0.634	−0.32	0.52
Household with Elderly	0.06	0.12	0.51	0.609	−0.17	0.29
Take Emergency Supply∗	0.42	0.09	4.51	0.000	0.23	0.60
Constant	−1.59	0.97	−1.64	0.100	−3.48	0.31

∗Statistically significant	LL(Null) = −205.36			N = 306		
	LL(Model) = −173.00			Wald chi2 (17) = 48.31		
	df = 18			Prob > chi2 = 0.0001		
	AIC = 382.00; BIC = 449.02			Pseudo R2 = 0.158		

### 4.3 Number of items carried

The outcome variable used for this inquiry was based on respondents' self-reported total count of items included in their emergency kits out of the total of 12 items. The result reports the model to be statistically significant (p = 0.000) with increase in income reporting a lower likelihood to taking more items of the emergency kit during evacuation and all other significant variables reporting a greater likelihood of carrying more items (***see*** Table 6).

**Table 6:** Predictors of carrying total number of emergency kit items.

Number of Items Taken (outcome)	IRR	Std. err.	z	P > z	95 % conf. intl	
1. Awareness of Flash Floods	1.00	0.30	0.00	0.999	0.56	1.79
2. Income						
Between rs.5000–10,000	0.86	0.10	−1.23	0.220	0.68	1.09
Between rs.11,000–25,000	0.98	0.13	−0.11	0.909	0.76	1.28
Between rs.26,000–40,000∗	0.68	0.13	−2.01	0.044	0.47	0.99
Between rs.41,000–55,000	0.90	0.23	−0.42	0.672	0.54	1.49
Above rs. 56,000∗	0.43	0.14	−2.66	0.008	0.23	0.80
3. Education						
Less than 9th grade	1.03	0.14	0.20	0.845	0.78	1.35
10th pass SSc	1.12	0.17	0.76	0.448	0.83	1.52
12th pass HSC∗	1.41	0.24	2.00	0.045	1.01	1.98
Some college∗	2.18	0.59	2.91	0.004	1.29	3.70
Some vocational certificate	2.04	1.48	0.99	0.324	0.49	8.42
Bachelor's degree∗	2.11	0.29	5.38	0.000	1.61	2.77
Grad./professional degree∗	1.52	0.30	2.13	0.033	1.03	2.23
4. Emotional types						
Positive Emotions∗	1.04	0.02	1.99	0.047	1.00	1.08
Negative Emotions∗	1.07	0.03	2.77	0.006	1.02	1.12
Perception of Risk∗	1.10	0.07	1.68	0.093	0.98	1.24
Household with special needs∗	1.39	0.15	3.00	0.003	1.12	1.72
Household with Elderly	1.11	0.08	1.51	0.131	0.97	1.28
Take Emergency Supply∗	1.29	0.04	7.74	0.000	1.21	1.38
Constant	0.20	0.09	−3.65	0.000	0.08	0.47

∗Statistically significant	LL(Null) = −553.01			N = 311		
AIC = 1087.5; BIC = 1166.02	LL(Model) = −522.74			LR Chi2(19) = 60.54		
Pseudo R2 = 0.055	df = 21			Prob > Chi2 = 0.000		

Specifically, increase in income, both medium (Rs. 26–40K) and high (over Rs. 56K), report a decrease in possibility of taking more emergency kit items by a factor of 0.68 (p < 0.05) and 0.43 (p < 0.01) respectively. Conversely, higher education tends to increase the likelihood of individuals taking more items of the emergency kit. For instance, individuals with HSC and graduate degrees reported an increase by the rate of about 1.5 times (p < 0.05) indicating an increase of 41 %–52 % respectively in the expected number of items taken compared to the baseline group. Those with some college or bachelors’ degree reported an increase by the rate of 2 times (p < 0.01) to carry more items of the emergency kit. These findings suggest that the likelihood of taking more emergency kit items increases by a factor of 1.5–2 times for individuals with higher education. The 95 % confidence interval gives the range where the true effect size is likely to lie, with 95 % certainty. The effect size is not significant for the intervals includes 1 for IRRs. While income reports a range below 1, confirming a significant negative effect on the number of items taken, the range of education variables confirms a positive effect on the number of items taken.

Interestingly, both positive (p < 0.05) and negative (p < 0.01) emotions increase the likelihood of taking more number of emergency items (by factor of 1.04 and 1.07 respectively) and risk perception increases the likelihood of taking more items (by factor of 1.10; p < 0.10). Not surprisingly both households with special needs members, and those with higher self-awareness of what is an emergency supply kit, demonstrated an increased likelihood of taking more number of items by a factor of 1.39 times (p < 0.01)and 1.29 times (p < 0.00) respectively. Barring risk perception that report a significance at p < 0.10, all other statistically significant variables report strong evidence against the null hypothesis with p < 0.05 or less. Also, the positive and negative emotions, household with special needs and taking emergency supplies report a strong positive effect on the number of items taken, with IRR values above 1. Perception of risk (CI: 0.98,1.24) reports a weak effect on the number of items taken during evacuation.

## 5 Discussions

This study investigated the household preparedness in a sort-of ‘ground zero’ situation wherein individuals surveyed for this study had no prior experience of flashfloods and almost all (98 %) were not aware of how to prepare for such incidents. Given that, it is encouraging to observe that at least 18 % of the respondents were aware that they were carrying emergency supplies with them during evacuation. This also emphasizes the need to raise awareness amongst the other 82%who did not, on the importance household preparedness to mitigate against future threats of flash-flooding. This is vital because while even those who responded not taking any emergency supplies did carry one or more of them but just not enough of them to sustain them for the first 72 h.

For instance, of those who said they did not carry an emergency supply, over 37 % of them carried a cellphone/radio, and over 22 % carried address of a safe location to evacuate. Other emergency kit items ranged from 0.8 % (important documents) to 17.4 % (cash or savings). Increasing awareness of the households and educating them on what constitutes an emergency supply kit is therefore imperative for them to prepare well for future incidents of flash-flooding in the region. In response to this, the premier disaster management entity in India, the National Disaster Management Authority (NDMA), has developed a list of emergency kit items for eight specific hazard types including floods, landslides, earthquakes, and urban floods [9]. While the list of emergency kit items for floods includes most of the items listed in this study, some unique items that the respondents carried with them included the LPG gas cylinder and blankets. This is unique to the climatic conditions of the mountainous region and populations surveyed, who acknowledged that lighting wood fires for cooking would be difficult during the cold wet months.

However, it also emphasizes the need to include the local populations in preparedness and evacuation planning through “Anticipatory Actions” (AA). These are defined as *acting ahead of predicted hazards to prevent or reduce acute humanitarian impacts before they fully unfold* [33]. Such actions are predicted to maximize the window of opportunity to trigger effective interventions to prevent or mitigate imminent impact on risk-prone communities and include prepositioning of financial incentives and allocations beforehand to ensure that post-disaster recovery is faster and smoother with local inputs.

For those who carried an or any emergency kit item versus those who did not carry any (Table 5, Table 6), it is important to understand the impact of the barriers that influence their decision to carry these items. The regression results revealed that higher educational attainment levels (bachelor and graduate degrees) positively influence their decision to carry emergency supplies. This is in line with findings by Ref. [26], who suggested that higher levels of education can mean a better understanding of risk, which can lead to more proactive preparedness behaviors. However, people with higher income were less inclined to carry any emergency supplies which requires further examination as it is contrary to other studies that suggest higher income levels enable greater access to resources such as emergency supplies, homeowners insurance, and transportation, translating into better disaster preparedness measures [34].

A seminal study by Zamboni and Martin [35] of 16,725 US households, suggested that 68.9 % of households fulfilled at least half of the recommended preparedness items, but households were more likely to fulfil resource-based items such as having an emergency carry-on kit and food and water stockpiles (resources), than alternative communication plans and meeting locations (actions). Furthermore, the wealthy and households with members over 65 were more likely to take most of these actions. These studies suggest that acknowledging the influences of income and education factors in disaster preparedness in these mountain communities will help policymakers craft targeted interventions. This is essential in vulnerable communities to reduce disparities and enhance resilience [36].

It is interesting to observe that both emotions reported a positive relation to taking emergency supplies, albeit only negative emotions reported a significant relation with the decision to take an emergency kit item. This is partly in line with Kasperson et al.’s [23] work suggesting that positive emotions can enhance collective resilience, while negative emotions can lead to panic. Emotions such as fear and anxiety can play a crucial role in motivating individuals and communities to engage in disaster preparedness activities. Slovic (2000) also suggested that fear of potential harm can lead to increased awareness and proactive measures. Positive emotions can motivate households to take proactive measures such as creating an emergency kit, create emergency plans, and increase social bonding that can help them better cope with stress and uncertainty [[37], [38], [39]].

Perceptions of risk reported a moderate significance (p < 0.10) yet positive relation with both choosing to take an emergency kit item and number of items. This is consistent with literature that underestimation of risk leads to lack of preparedness [26], while individuals who perceive a higher level of risk are more likely to engage in preparedness activities such as creating emergency kits and developing evacuation plans [40].

Households with special needs reported a significant positive relation with number of items taken but not with the decision to take an emergency kit item or not indicating the higher the number of individuals with special need, the likelihood of taking more items increased by 1.39 times. However, further investigation revealed about 47 % of these household took cell phone/radio and address of safe location followed by cash and clothes (28 %). Households with special needs are recommended to maintain a sufficient supply of essential medications, medical equipment, and assistive devices in their emergency kits [41]. Considering only 11 % carried medicines with them is worrying and, indicates a need to raise awareness and preparedness of such households to carry a first aid kit, as well as medicines for chronic illnesses, and life-threatening diseases for the elderly, and medicines for children.

Self-awareness of taking an emergency supply reported a positive relation with both, the decision to take emergency kit items and the number of items. Even with no prior experience of flash-flooding and less awareness training/education, the decision to take more items of the emergency kits increased by 1.29 times. Awareness training and support for community involvement in village and household preparedness initiatives can only enhance their decision making and educate those unaware to be more prepared for future flash-flooding event in the region.

## 6 Conclusions

This study examined the preparedness of households to flash-flooding in the mountainous region of Uttarakhand, India. With none of the respondents having experienced a flash-flooding before, and almost none having attended any awareness sessions about preparedness to flooding, this study presents a ‘ground-zero’ like situation to understand household preparedness to flash-flooding. We examine the self-awareness of these individuals, their decision to take emergency kit item and the determinants of how many items they chose to take during evacuation. With hardly any respondents aware of how to prepare for such event, higher income, higher education levels, emotional intelligence, and higher perception of risk can be the key determining factors for individuals to select one or more emergency kit items during evacuation. However, improved awareness and support can enhance the preparedness of the communities against future floodings.

This study provides important insights into the decision-making behavior that impacts the preparedness of communities in response to future flooding in Uttarakhand. However, this study uses responses from a those affected by flooding in 2013. A longitudinal assessment of the same can provide insights into the enhancement in preparedness that has resulted since 2013. A present no statistical factsheet is available to assess the current investment by local and non-governmental agencies in providing emergency kits of the residents of the region. Such assessment is needed to understand the reach of awareness and support available to those potentially impacted by future events. Also, this is one of very few studies that examines the efficacy of the households with regards to emergency kits. Similar studies are needed to understand the reasons why communities are under-prepared and what action-based approaches are required to enhance the resiliency of the communities subjected to flash floods globally.

Several respondents were unaware that they were indeed carrying an emergency supply and even those who were aware, did not carry some of the essential items in an emergency kit such as medicines and important documents. Educating them about the essentials of an emergency kit can prepare the communities better for flooding in future. Since this study in 2013, the NDMA has created a list of essential items for eight kinds of natural disasters including flooding and landslide. While majority of the items in both, some items such as a Global Positioning System (GPS) (e.g. one in a smartphone), are recommended for landslides but not for flooding. This can be confusing for at-risk populations prone to cascading threats such as flash-flooding leading to landslides and susceptible to earthquakes as well, as the case of the state of Uttarkhand. The NDMA could recommend the items to be carried for multi-hazards in areas of cascading threats, to better prepare the community to respond to the situations.

Furthermore, the anticipatory actions approach can be effective in educating the population prior to the risk and help them prepare an emergency kit in these multi-hazard conditions in future. However, the digital map by the United Nations Office for Coordination of Humanitarian Affairs (OCHA) displays nations that are part of the Anticipatory Actions initiatives and at present, India is still not part of their portfolio. Becoming a participant and taking such proactive approaches can aid NDMA for better preparedness to flooding and landslides in mountainous regions of Uttarakhand. Adapting the anticipatory action framework in the Indian context should be given serious consideration by appropriate local and national disaster management authorities in India.

High-income households have reported better preparedness to disasters However, the mediator factor is the awareness to disasters. High-income communities report better preparedness when they report higher awareness to any disaster [42]. However, with little to no awareness, even high-income communities report low preparedness to disasters [43]. This study reported little to no-awareness, irrespective of the income levels, indicating that increased awareness is essential for advanced preparedness and effective response at household level to flooding.

Emotional resilience, which involves the ability to cope with and recover from the emotional impacts of disasters, is a critical aspect of disaster preparedness. Norris et al. [44], reported that education and mental health support can enhance emotional resilience. Hence, the local authorities should increase the availability and awareness about the importance of post-intervention techniques and encourage the affected population through psychological support to reduce anxiety and feeling of unnecessary fear, hopelessness or helplessness [45]. Also, community-based approaches through education and health support from local and national governmental authorities can help the victims in their recovery [46].

Households with special needs can play an active role in advocating for inclusive emergency planning and policies at the local and national levels. Participating in community emergency planning committees and engaging with policymakers can lead to positive change [2]. Future anticipatory research and preparedness should target such households to include their needs and barriers to recommend a comprehensive kit for multi-hazards in the region of Uttarakhand.

We acknowledge that this study was conducted 10 years ago, and the State Government of Uttarakhand has made great strides in preparedness activities which we have not been able to review. Despite this, our findings help to underscore:

1. The need to ensure disaster kits are recommended by every state/district and Panchayat (local) levels for residents and in communities with transient populations like visitors/tourists they are distributed at points of entry.
2. Private sector agencies are invited to help stock various items in these kits as part of their Corporate Social Responsibility efforts.
3. Identify at risk populations and teach them how to build disaster kits and revive the National School Safety Programme (NSSP) to help school children create disaster preparedness kits (NDMA, b).
4. Conduct additional empirical research in various disaster contexts to demonstrate the benefits of disaster preparedness kits for immediate survival and resilience.

## CRediT authorship contribution statement

**Praveen Maghelal:** Writing – review & editing, Writing – original draft, Visualization, Methodology, Formal analysis, Conceptualization. **Sudha Arlikatti:** Writing – review & editing, Resources, Investigation, Funding acquisition.

## Data availability statement

The datasets generated during and/or analysed during the current study are available from the corresponding author on reasonable request.

## Funding

This work was supported by a grant from the US National Science Foundation (RAPID Grant Award No. 1361323). Any opinions, findings and conclusions or recommendations expressed in this material are those of the authors and do not necessarily reflect the views of the National Science Foundation.

## Declaration of competing interest

The authors declare that they have no known competing financial interests or personal relationships that could have appeared to influence the work reported in this paper.
