# Supplementary material for: Disaster preparedness kits ready or not? Household resilience to flash flooding in Uttarakhand
Source: Heliyon. 2024 Dec 26;11(1):e41446. doi: 10.1016/j.heliyon.2024.e41446 (PMC11755034; doi:10.1016/j.heliyon.2024.e41446)
Supplement: Multimedia component 1 [file mmc1.pdf]

IMMEDIATE BEHAVIORAL RESPONSE TO THE FLASH-FLOODS IN UTTARAKHAND, NORTH INDIA

UNIVERSITY OF NORTH TEXAS

Restricted

IMMEDIATE BEHAVIORAL RESPONSE TO FLASH-FLOODS IN UTTARAKHAND, NORTH INDIA

Village Name/ Location: ______________________________ Date/ Interviewer: _____________________________

1. When the 17th June 2013 flash flood occurred, in which community (village) were you located?

2. When the flash flood occurred, which of the following best describes where you were? (Tick ***only one***)

_1__ At home
__2__ At a friend or relative's home
_3__ At my workplace
__4__ In transit, walking to/from work or shopping etc.
_5__ At the temple
__6__ In a public place
_7__ In a vehicle
__8__ Other (please explain):_____________________________________

3. When the flash flood occurred, which of the following best describes the location of other members of your family?

_1__ All individuals were together at home
_2__Some household members were absent but I knew they were in a safe location
_3__ Some household members were absent and I knew they were in danger
_4__ Some household members were absent and I did not know if they were safe

4. When the flash flood occurred, what was your social context? (Tick ***all that apply***)

_1_ I was alone
_2_ I was with children under 18 years of age
_3_ I was with adults I knew
_4_ I was with adult strangers

5. How strong were the sounds of the flash flood to you?

_1_ Unnoticeable
_2_Weak
_3__ Mild
_4_Moderate strength
_5_ Strong
_6__ Violent

6. What was your first response while the flash flood occurred? (Tick ***only one***)

_1_ Continued what I was doing before the flood
_2_ Stopped what I was doing but stayed where I was
_3_ Stopped what I was doing and climbed to higher level and held on to the furniture
_4_ Tried to protect other people nearby
_5_ Tried to protect property nearby from the water by lifting it
_6_ Immediately left the building I was in
_7_ Continued driving
_8_ Pulled over to the side of the road
_9_ Other (please explain) ____

7. To what extent did you feel each of the following emotions during the floods?	**Not at <u>All</u>**		**Very great <u>extent</u>**
a. optimistic	1	2	3
b. depressed	1	2	3
c. annoyed	1	2	3
d. nervous	1	2	3
e. fearful	1	2	3
f. relaxed	1	2	3
g. energetic	1	2	3
h. alert	1	2	3
i. passive	1	2	3

Restricted

8. Who provided you information **firs**t about the flash flood and what to do? (Tick ***only one***)

_1__ Village leaders (panchayat members, priest etc.)   _2__ Friend, relative, neighbor, or co-worker
_3__ Radio/TV Announcer   _4__ Police
_5__ Villager   _6__Self

9. To what extent did you believe the flash flood would do each of the following…	**Not at all**		**Very great extent**
a. severely damage or destroy your home?	1	2	3
b. injure or kill you or your family?	1	2	3
c. disrupt your job and prevent you from working?	1	2	3
d. disrupt electrical, telephone, and other basic services?	1	2	3
e. destroy or severely damage many homes in your village/town?	1	2	3
f. injure or kill many people in your village if they did not evacuate?	1	2	3
g. place your life and the lives of your family at risk if you did not take precautionary measures and evacuate from where you were?	1	2	3

10. What was your **first** source of information about the flash flood? (Tick ***only one***)

_1__ I was aware that the torrential rains could cause a flash flood (please go to Q12).

_2__I saw animals behaving unusually (What type of animals? ______________________Proceed to Q12).
_3__ I noticed unusual changes in the mountain slopes and around me (proceed to Q12)
_4__ I saw many people begin to evacuate (proceed to Q12)
_5__ I saw the flash flood coming (proceed to Q12)
_6__ I was warned by a village bell ringing about the flash flood (proceed to Q12)
_7__ Others warned me face to face about the possibility of a flash flood (proceed to Q10)
_8__ Someone called or texted to warn me about the flash flood in other villages (proceed to Q10)
_9__ I heard a warning from a radio/TV source (What channel or radio station? ________Proceed to Q10)
_10__ Other: ____(proceed to Q 10)

11. What was the content of the information you received from that first source? (Tick ***all that apply***)

_1_ What the threat was (e.g. flash flood/ land slide)   _2_ Warning applicable to certain areas
_3_ Recommended actions (e.g, leave your home)   _4_ Safe locations/where to go
_5_ Where assistance can be received from   _6_ Where further information can be found
_7_Other: ____

12. After you received the first information regarding what to do about the flash flood, from which **other** sources did you receive information?

_1_ Saw the flash flood approaching   _2_Witnessed people evacuating
_3_ Others provided info face to face   _4_ Received info via cell phone call
_5_ Received info via radio   _6_ Received info via TV
_7_ Received info via text message   _8_Received info via email
_9_Received info via siren   _10_Received info via village bell
_11_Other: ____

13. When the flash flood struck, how certain were you a landslide was imminent?

Not certain 1   2   3   4   5 Very certain

Not certain 1 → Go to Q14
2–5 → Go to Q13

14. When the flash flood struck, how long did you think it would take before there would be a landslide?

______ hours/Minutes

Restricted

15. Were you advised/warned by anyone to evacuate to safer location? (Tick ***all that apply***)
   _1_ No _2_ Yes, notified by an authority (e.g., Village Chief)
   _3_ Yes, notified by news media _4_Yes, notified by friends, neighbors/relatives
   _5_ Tourist

16. Did you try to evacuate your family from the location you were at when the flash flood struck? (Tick ***only on***e)
   _1__ Yes, we evacuated uphill away from the water (proceed to Q16)
   _2__ Yes, we evacuated down-hill away from the water (proceed to Q16)
   _3__ Yes , we evacuated horizontally inland away from the water (proceed to Q16)
   _4__ Yes, we evacuated to the second or higher story of a structure (proceed to Q21)
   _5__ No, I stayed where I was and continued what I was doing (proceed to Q24)
   _6__ No, I stayed where I was and awaited further warning or info (proceed to Q24)
   _7__ No, I went to watch the incoming waters (proceed to Q24)
   _8__ No, I took some other action (describe): ________________________________(proceed to Q24)

17. At the time you evacuated from your home/location, did you have a personal vehicle you could use to evacuate?
   __1___ No __2__yes

18. How did you evacuate from your location? (Tick ***all that apply***)
   _1_By foot _2_My vehicle _3_ Relative/Friend vehicle _4_ Public transportation (bus, taxi, etc.)
   _5_ Emergency Vehicle _6_ Other (please explain): ____

19. When you evacuated, did you head towards a different village?
   _1__ No (proceed to Q20) _2__Yes (proceed to Q19)

20. To which village did you go? ____

21. To which of these locations did you head to once you had evacuated from your home? (Tick ***all that apply***)
   _1_Relative's home _2_Friend's home _3_Official Government Shelter
   _4_ Open public space (e.g., Park, mountain slopes) _5_Nonprofit Shelter
   _6_Temple/ religious building _7_ Public building (e.g. school, marriage hall, govt. building)
   _8_ Other (please explain): ____

22. How long did you wait after the flash flood stopped before you evacuated? ______ minute(s)

23. Did you try to find additional information before you evacuated? (Tick ***all that apply***)
   _1_ No _2_Yes, from friends/ family/neighbors _3_ Yes from leaders (church, Village Chief, etc.)
   _4_ Yes, from news media _5_ Yes, from other tourists
   _6_Yes, other (please explain): ____

24. What action(s) did you take before you evacuated from your location? (Tick ***all that apply***)
___ Looked for separated family members ___ Secured my property
___ Gathered some emergency supplies ___ Warned others
___ Other (please explain): ____

25. Were you caught in the flash flood? __1___ No ___0___ Yes

26. Which of these materials did you have at hand when the flash flood hit? (Tick ***all that apply***)
_1_Supply of water. How much?____
_2_Supply of non-perishable foods? What kind, how much? ____
_3_Medicines? What kinds and how much? ____
_4_Cash or other forms of savings? ____
_5_A change of clothes? How many days? ____
_6_Female hygiene products? ____
_7_Cell phone/ battery operated radio etc? ____

Restricted

_8_Address of safe zone/location ____
_9_Other (explain): ____

27. Have you experienced a flash flood before the June 17th one?
 _1_No _0_Yes

28. If 'Yes' then how many times in the past ten years? ___________ (number of times)

29. Did you have to evacuate for any of those floods?
 _1_No _0_Yes

30. To protect yourself and your family from future flash flood risks, which of these actions or measures will you take different from what you did took this year?

	**Not at all**		**Very great extent**
a. Look for additional information about flash floods?	1	2	3
b. Have a family emergency plan?	1	2	3
c. Participate in discussions with neighbors and village leaders?	1	2	3
d. Work to create a village emergency plan?	1	2	3
e. Move during the monsoon season?	1	2	3
f. Relocate permanently to another location?	1	2	3
g. Build stronger homes/ raise the home?	1	2	3
h. Watch for environmental cues?	1	2	3
i. Other (explain): ____________________	1	2	3

31. How old are you? _______ years

32. What sex are you? _1_ Male _2_ Female

33. Marital status: _1_Married _2_ Single _3_Divorced _4_Widowed

34. How many members of your family are:
 Below the age of 18?_______ Between 18 and 65? _______ Over 65 years of age _____

35. Did any member of your household have special needs and require assistance to evacuate?
 __No __Yes If 'Yes' Explain:____

36. What caste do you belong to,
 __1___ Upper Caste _2__Backward Caste _3__Most Backward Caste _4__Scheduled caste/tribes
 Other (please explain): ________________

37. What is your religion? _1_ Hindu _2_Muslim _3_Christian _4_Buddhist _5_Other :_______________

38. What is the highest education level you have completed? (Tick ***only one***)
 _1_ Illiterate _2_Less than 9th grade _3_10th pass (Secondary School Certificate)
 _4_12th pass (Higher Secondary School certificate) _5_ Some college, no degree
 _5_ Some certificate _6_Bachelor degree _7_ Graduate or professional degree

39. What is your family's monthly income range?
 __1_Below Rs. 4,999 _2_ Between Rs. 5,000 to 10,000 _3_ Between Rs. 11,000 to 25,000
 _4_ Between Rs. 26,000 to Rs. 40,000 _5_Between Rs. 41,000 to 55,000
 _6_Above Rs. 56,000 _7_ Other (no income Explain): _________________________________

40. Do you rent or do you own the home you live in? _1_ Rent _2_ Own Home

Restricted

41. How long have you lived in the village where you now reside? _______ Years ______ Months

42. Before the flash flood and landslides on June 16-17th 2013 had you ever attended any…
   a. flash flood hazard awareness meetings? _1_ No   _0_ Yes
   b. landslides hazard awareness meetings? _1_No   _0_Yes

43. Before the flash flood and landslides on June 16-17th 2013 had you ever received any…
   a. flash flood hazard awareness brochures ?   _1_No _0_ Yes
   b. landslides hazard awareness brochures?   _1_ No _0_ Yes

44. Was any member of your family killed or injured,
   a. by the flash flood?   _1_ No _0_ Yes
   b. by a collapsed building? _1_ No _0_ Yes
   c. by other causes? Explain: ____

45. Is any member of your family still missing?
   _1__ No _2__ Yes

46. How would you rate the damage to your home/place of residence by the flash flood and landslide?
   _1_ No damage   _2_ Minor or slight damage   _3_ Moderate damage
   _4_Extensive or severe damage   _5_ Totally destroyed

47. To protect yourself and your family from future flash floods and landslides, will you be relocating your home from your present village to another village?
   _1_ No (proceed to Q.48)   _0__ Yes

48. If Yes, then where to? (Other village, Dehradun/ Delhi, other state, lower plains etc.)
____

49. If no, will you take special protection during the monsoon months?
   _1__ No   _0__ Yes

If yes, what actions will you take, please explain?
____
____
____

50. Do you have any further comments you would like to make regarding you and/or your family's experience of the June 16-17th flash flood disaster? Please note both positive and/or negative.
a. Positive:
____
____
____
____
____
____

b. Negative:
____
____
____
____
____

Thank you for participating in this survey!

Restricted
